# Correlation between Cutting Clearance, Deformation Texture, and Magnetic Loss Prediction in Non-Oriented Electrical Steels

**DOI:** 10.3390/ma14226893

**Published:** 2021-11-15

**Authors:** Ján Füzer, Samuel Dobák, Ivan Petryshynets, Peter Kollár, František Kováč, Ján Slota

**Affiliations:** 1 Institute of Physics, Faculty of Science, Pavol Jozef Šafárik University in Košice, Park Angelinum 9, 04154 Košice, Slovakia; samuel.dobak@upjs.sk (S.D.); peter.kollar@upjs.sk (P.K.); 2 Institute of Materials Research, Slovak Academy of Sciences, Watsonova 47, 04001 Košice, Slovakia; ipetryshynets@saske.sk (I.P.); fkovac@saske.sk (F.K.); 3 Department of Computer Support of Technology, Faculty of Mechanical Engineering, Technical University of Košice, Mäsiarska 74, 04001 Košice, Slovakia; jan.slota@tuke.sk

**Keywords:** electrical steels, punching, permeability, energy loss

## Abstract

Manufacturing the magnetic cores in electrical machines impacts the magnetic performance of the electrical steel by inducing stresses near the cutting edge. In this paper, energy loss behaviour in non-oriented electrical steels punched with different cutting clearances before and after annealing is investigated. An experimental shear cutting tool was employed to punch the ring-shaped parts from electrical steels in a finished state with four different values of cutting clearance corresponding to 1%, 3%, 5%, and 7% of the sheet thickness. The effect of cutting clearance on the magnetic losses is derived and analysed by the statistical theory of losses and associated loss separation concept including the analysis of movable magnetic objects. In this framework, this paper assesses the combined effect of cutting clearance, frequency, and heat treatment on the hysteresis loops and iron losses in non-oriented FeSi electrical steels. Measurements have been performed from quasi-static to 400 Hz at peak induction *B*_p_ = 1.0 T. Both states before and after heat treatment have been considered. The excess loss is observed as the most sensitive loss component to cutting clearance and its magneto–structural correlation is quantified.

## 1. Introduction

The laminated cores made of non-oriented (NO) electrical steels are the prime materials employed in transformers and electric rotating machines. As the magnetic loss covers about 5–10% of the generated electric power [1], there has been an increasing interest in the development of advanced preparation process to reduce the core loss by the optimization of purification [2], rolling [3], additive manufacturing [4], and grain texture recrystallization [5,6]. An inevitable step to fabricate the steel parts of proper geometry is their cutting which, however, induces specific deformation textures and stress patterns [7], in particular close to the cutting edge. This can lead to the significant deterioration of magnetic properties through the inhibition of domain wall motion [7]. Although substantial efforts have been devoted to the investigation of manifold cutting tools—e.g., punching [8], laser [9], photo-corrosion [10], water-jet [11], spark-erosion [12], etc.—the main industrial techniques are mechanical punching and laser cutting. The former is routinely applied in mass production while the latter is for the newly designed prototypes only [13]. Paltanea et al. [14] developed a phenomenological model to determine the region impacted from a magnetic viewpoint. They revealed that cutting can affect a region that extends up to 4 mm into the electric steel strip. The associated mechanism of the deterioration of magnetic properties at the atomic level is the change in inter-atomic distances and related variation in the band structure. That causes changes in the coupling of magnetic moments in the mechanically deformed zones. However, magnetic properties can be restored via stress relief annealing [15,16].

Among the works on the processing of electrical steels by means of cutting, attention is given mainly to the influence of the cutting procedure (e.g., mechanical, laser, water) on magnetic behaviour [17,18,19]. Previous approaches to identify the characteristics of the cutting process play a crucial role in the optimization of the sheared edge quality investigated the links between various mechanical and geometrical properties such as the burr height, the wear of the cutting tool, and the thickness of a sheet [20,21]. Far fewer studies focus on the correlation between cutting clearance and magnetic properties in non-oriented electrical steels. The main technical parameter controlling the shear cutting is the cutting clearance—i.e., the width of a gap between the punch and die—expressed in the percentage of the lamination thickness. Cutting clearance is an important processing factor, mainly responsible for the cutting edge quality and thus it has a great influence on magnetic property deterioration. According to Baudoin et al. [22], there is a transition zone corresponding to the drastic change of the knife displacement at fracture. The existence of a transition zone was shown in the range of applied clearances, for which the magnetic properties were severely worsened. Subramonian et al. [23] developed a method to select a geometry dependent variable punch-die clearance to achieve more uniform wear on the punch, thereby enhancing the punch and die life by minimizing tool wear. Cao et al. [24] measured the residual stress after the punching process. The residual stress affected the zone of punching by about 0.4–0.5 mm in width. The sample before annealing exhibited complex domain patterns and the widths of the magnetic domains varied between 3 μm and 8 μm. Most of the domain walls in the annealed sample were of 180° and 90° type, and the widths of the domains decreased to 1–3 μm. The experiments by Weiss et al. [25] show that high cutting speeds lead to a larger strain-hardened area, which, in turn, gives rise to higher residual stresses and magnetic losses. The finite element analysis demonstrated that smaller cutting clearances may lead to higher residual stresses close to the cutting surface, but they also reduce the penetration depth. Wang et al. [26] studied the influence of grain size and punch-die clearance on the magnetic properties worsening in the blanking the thin non-oriented electrical steel sheets. They suggested that the optimal cutting clearance depends on the mean grain size 〈*s*〉 as ∝ 1/〈*s*〉^1/2^ and about 6% magnetic loss can be reduced by optimization of the blanking clearance, which is highly beneficial for energy saving. Hambli [27] proposed the fast modelling the edge formation using anartificial neural network. Recent finite element simulations have led to the description of sheared edge morphology in terms of crack propagation [28]. Nevertheless, there is a lack in the literature concerning the effect of the cutting clearance on the magnetic properties of non-oriented electrical steels, particularly in relation to the mechanical characteristics of the deformation process and magnetization processes in the medium frequency range. Despite the number of experimental and numerical studies of the cutting dynamics in electrical steel laminations shedding light on the qualitative aspects of fabrication process.

The motivation of this work was the accurate and efficient prediction of the magnetic loss over frequency in conjunction with the quantitative assessment of the cutting effect on the intrinsic nature of magnetization process being conspicuously absent in the literature. Precise loss modelling is key to the proper simulation of efficiency and to the fault prevention in electric machines. In this paper, we focus on the systematic study of the influence of mechanical punching before recrystallization annealing on the distributed micro-hardness and its correlation to the magnetic domain behaviour translated into experimentally determined and theoretically predicted core loss components as a function of cutting clearance.

## 2. Materials and Methods

**Figure 1:**
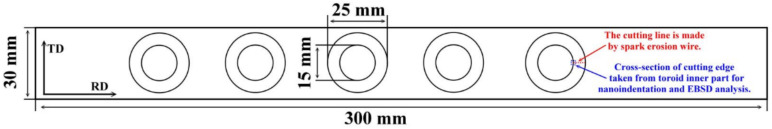
Scheme of ring samples cutting. TD = transverse rolling direction, RD = rolling direction, EBSD = electron back-scattered diffraction.

The ring samples of outer and inner radii of 25 mm and 15 mm, respectively, were cut from *d* = 0.5 mm thick vacuum degassed fully finished NO Fe–Si sheets (M350-50A grade) by punching using the shear-cutting tool with changing cutting clearance of 1%, 3%, 5%, and 7% of *d*. The ring samples were cut from the steel sheets with planar dimensions 300 mm × 30 mm. The schematic overview of experimental cutting procedures is displayed in Figure 1.

The chemical composition of the precursor sheet is (in wt.%) Fe = 96.8, C = 0.003, Si = 2.42, Mn = 0.268, P = 0.014, Al = 0.398, other elements ~0.097. Microstructural analysis was performed by the light optical microscope OLYMPUS GX71 (OLYMPUS Europa Holding GmbH, Hamburg, Germany). In order to obtain the distribution of grain size near the cutting edge, the selected microstructural states were processed by using free metallographic analysis software ImageJ (version 1.46, National Institutes of Health, Bethesda, MD, USA). It enabled us to estimate the average grain size of the samples after the heat treatments. Due to the irregular shape of grains, the average grain size was calculated as the Feret mean diameter [29] and via ImageJ. The texture analysis was carried out by means of the electron back-scattered diffraction (EBSD) method in the normal direction plane. The scanning electron microscope JEOL JSM 7000F (Jeol Ltd., Tokyo, Japan) with the EBSD detector Nordlys-I (HKL technology A/S, Hobro, Denmark) were used to perform the texture analysis. The obtained EBSD data were processed by the CHANNEL-5, HKL software package (Service pack 7). For individual analyses (i.e., microstructure, texture, nanoindentation, and magnetic measurement analyses), experimental samples were heat treated in laboratory conditions using the electric resistance furnace Nabertherm RS 120/1000/13 (Nabertherm GmbH, Lilienthal, Germany). The long-term annealing treatment was carried out according to the EN 10 106 standard [30]. The samples were slowly heated up to 790 °C at a heating rate of about 100 °C/h. Subsequently, they were held at the temperature of 790 °C for 1 h and finally cooled at a rate of 200 °C/h. The annealing atmosphere was pure hydrogen with a dew point of 25 °C. The heating and cooling rates of the conventional long-term annealing process were controlled by internal thermocouples operated by digital controller of the furnace. The microindentation measurement of the local hardness was carried out Agilent G200 indentor (Agilent Technologies, Inc., Chandler, AZ, USA) with a Berkovich tip perpendicular to the cutting surface mapping the matrix of points with 20 μm spacing. Measurements were done using single loading–unloading indentation. In all cases, an indentation depth-controlled method was used with a maximum depth of 500 nm. The electrical conductivity in the investigated Fe–Si steel, measured by a standard four-point probe method, is 23 × 10^5^ Ω^−1^m^−1^. The magnetic measurements were performed by means of a digital hysteresisgraph AMH-1K-S according to the IEC 60404-6 standard. Cross-sections of samples used for nanoindentation and EBSD analysis were prepared by electrical discharge machining using spark erosion machine EIR-EMO 2N (Emotek s.r.o., Nové Mesto nad Váhom, Slovakia). The cutting line was carried out on the ring samples along the rolling direction, see Figure 1.

## 3. Results and Discussion

### 3.1. Microstructure

**Figure 2:**
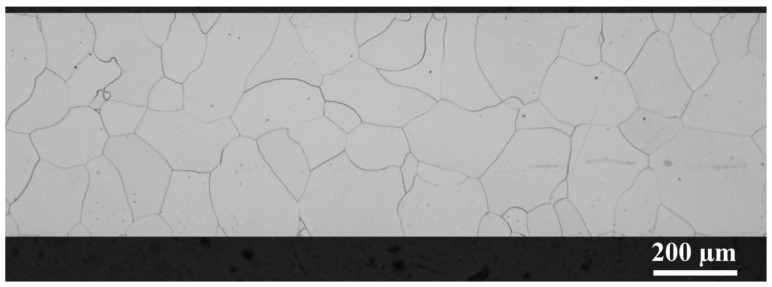
Initial (as-received) microstructure of investigated ‘fully finished’ non-oriented electrical steels before punching and annealing.

Microstructure analysis of investigated steels was carried out by a light optical microscope. The initial microstructure of the ‘fully finished’ non-oriented electrical steels used to the punching of ring-shaped is presented in Figure 2. This light-optical microstructure observation was performed on metallographic cross-sections of individually selected sample with their longitudinal part oriented parallel to the rolling direction. The optical micrograph clearly illustrates that samples are characterized by monophasic ferrite microstructure with homogeneous distribution of uniaxial polygonal grains. The performed image analyses indicated their average dimensions to be about 150 ± 17 µm.

**Figure 3:**
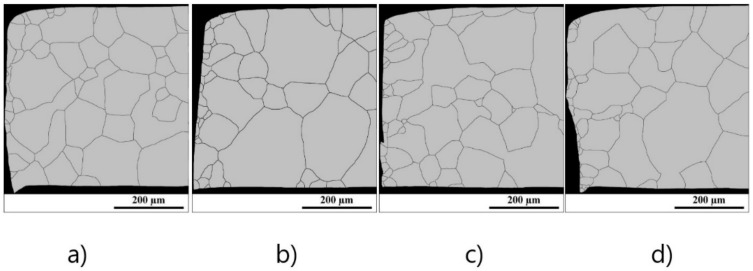
Microstructure of cutting surface cross-section observed after punching and annealing obtained by image analysis of rings with cutting clearances corresponding to 1% (**a**), 3% (**b**), 5% (**c**), 7% (**d**) of the sheet thickness.

**Figure 4:**
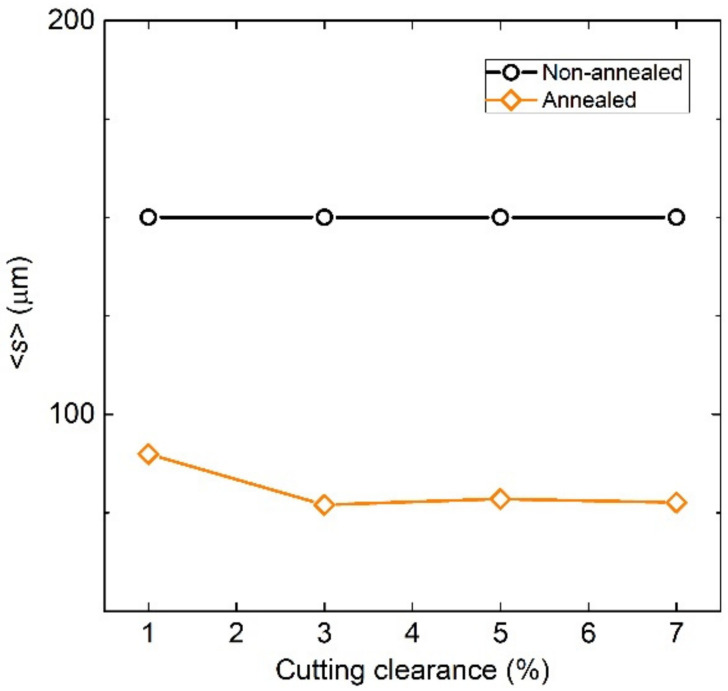
Comparison of mean grain size in non-annealed and annealed samples.

The microstructure evolution of experimental cut edges obtained with different clearances after the long-term annealing which was carried out according to the EN 10106 heat treatment is presented in Figure 3. The analysis of microstructure was performed on a metallographic cross-section along the rolling direction with an approximate distance of about 600 µm from the sheared edge. These results clearly demonstrate that presented samples were characterised by a microstructure matrix with bimodal distribution of ferritic grains. The fine-grained microstructure is detected mostly on the cut edge with an average grain size in the range of 15–25 µm. These parts of cutting samples consist of the high value of residual plastic mechanical stresses. Because of this, the heat treatment in static conditions allows achieving the primary recrystallization of ferritic grains only vicinity of the cutting edge. The coarse-grained microstructure is presented at a distance of approximately 50 µm from the cutting edge and the average grain size of these microstructures corresponds to the average grain size of microstructure calculated for the initial samples (see Figure 2). The average grain size for cutting edges presented in Figure 2 was obtained on the cross-section with dimension 500 µm × 600 µm, where 500 µm is the thickness of samples and 600 µm is the distance from the cutting edge. The image analysis shows that samples with cutting clearances 1%, 3%, 5%, and 7% have an average size of about 87.9 µm, 77.2 µm, 78.5 µm, and 77.6 µm, respectively. The comparison of average sizes of investigated samples in their individual material states is presented in Figure 4. This graph clearly demonstrates the effect of cutting clearances and heat treatment on the microstructure evolution near the cutting edge. It can be concluded that high-intensity mechanical deformations and long-annealing treatment lead to the refinement of microstructure in the vicinity of the cutting edge.

### 3.2. Texture and Local Misorientation Profile

The crystallographic texture of samples, as well as the local misorientation maps, are important parameters influencing the magnetic properties of non-oriented electrical steels in the final state and after the cutting. In order to characterize the evolution of local mechanical strains in the vicinity of the cutting edge and textural changes of the investigated samples with different cutting clearance, specific EBSD analyses were performed.

**Figure 5:**
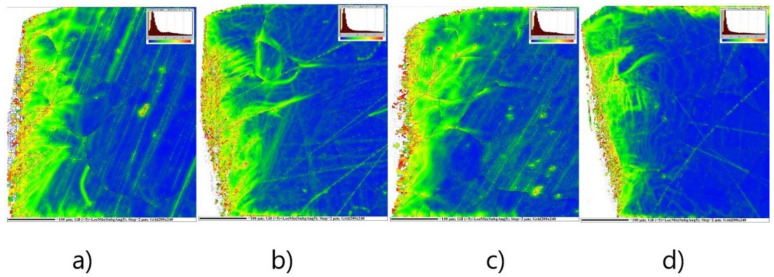
Local misorientation map of cutting surface cross-section of rings with cutting clearances corresponding to 1% (**a**), 3% (**b**), 5% (**c**), 7% (**d**) of the sheet thickness before annealing. The legend (the upper right corner) for angles ranging from 0–5° which are marked by rainbow colour and showing the higher deformation region.

The Figure 5 represents the average local misorientation map (LMM) obtained from the EBSD data. It illustrates where there is a high level of misorientation vicinity near the cutting edge before annealing. The high misorientation is an indication of the areas of mechanical strains which are related to plastic deformation. In other words, the value of local misorientation is related with the degree of deformation. Once the cutting process is imposed, the grains are forced to deform, which leads to the formation of dislocations. The accumulation of high dislocation density produces high misorientation in strongly deformed regions. This type of analysis (Figure 5) allows us to identify the large amount of plastic mechanical strains which are visualized in the microstructure around or inside investigated ferrite grains. Here, the emphasis is given to resolving the substructural boundaries that are typically misoriented by more than 2°. The legend (in the right corner) shows the intensity of misorientation angles on the analysed surface. It is clearly visible that the major portion (blue tone colours) of the coarsened grain matrix is characterized by a very low misorientation angle (around 0°). It indicates that these ferrite grains are not plastically deformed. Nevertheless, the micrograph in Figure 5 shows that the microstructural part vicinity of the cutting edge is characterized by a high level of misorientation angles in the range of 2–5° and even more. It indicates that these grains are characterized by increased intensity of mechanical strain associated with high dislocation density. Evidently, the width of deformed zone near the cutting edge depends on the cutting clearance. The results presented in Figure 5 clearly show that the depth of the penetration of residual stresses in the area of the cutting surface was significantly lower when the shear cutting process that was performed with a smaller cutting clearance. On the other hand, the local misorientation maps show that smaller cutting clearances may lead to higher residual stresses immediately next to the cutting surface. The results also show that a smaller cutting clearance reduces the size of the area affected by shearing.

**Figure 6:**
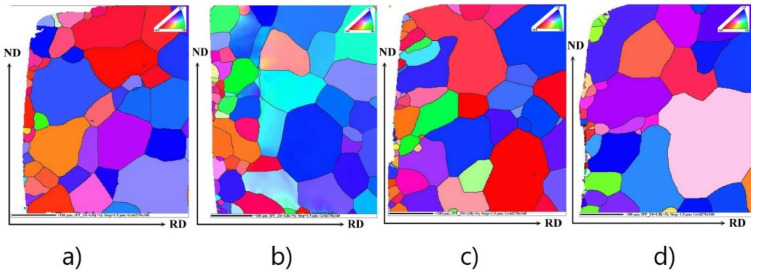
IPF maps present the characterization of the textural state near the cutting edge of rings with cutting clearances corresponding to 1% (**a**), 3% (**b**), 5% (**c**), 7% (**d**) of the sheet thickness after annealing. The colour key for the identification of crystallographic orientation of grain is located in the upper right corner of the IPF map.

The common texture of experimental steels near the cutting edge obtained from the rings after annealing with different cutting clearances is shown in Figure 6. The crystallographic orientation of matrix grains in the vicinity of the cutting edge of samples with different cutting clearances is presented as an inverse pole figure (IPF) map. The IPF visualization of microstructure provides complementary crystallographic characterization to the light-optical microstructure observation (Figure 3). The performed EBSD analyses demonstrate that the fine grains located near the cutting edge are characterized by Goss texture (green grains) or rotated cube {001} <110> (red grains) texture. The coarse-grained microstructure, which corresponds to microstructure in the initial samples, is represented mostly by the blue colour tone which is responsible for the strong deformation texture {111} <uvw> (see Figure 6). In the case of these results, it is not easy to discuss how the crystallographic texture of grains obtained after primary recrystallization influence the magnetic properties of samples with different cutting clearances. We can conclude only that fine-grained microstructure near the cutting edge is the most important structural parameter which could be responsible for increasing magnetic losses of samples with higher cutting clearance.

### 3.3. Nano Indentation Measurements

**Figure 7:**
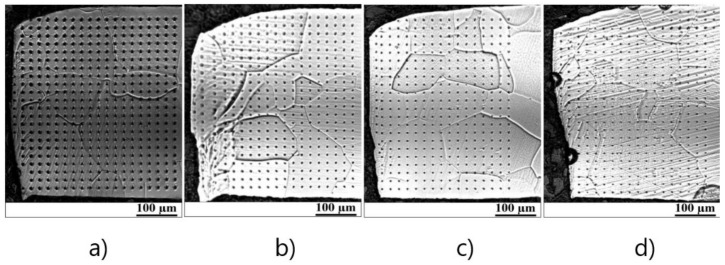
Grain structure in combination with a matrix of nanoindentation measurements carried out at vicinity of cutting edge of samples with (**a**) 1%, (**b**) 3%, (**c**) 5%, and (**d**) 7% of cutting clearance before annealing.

The different cutting clearance used for the experimental steels is responsible for the residual mechanical stress behaviour in the vicinity of the cutting edge. To investigate the value of plastic mechanical strains and their dependence on the distance from the cutting edge, the nano-hardness tests were carried out. It is well-known that steel, after plastic deformation, possesses accumulated deformation energy, which is stored in dislocation structures. It means that steel subjected to a cutting process is characterized by increased dislocation density, causing a certain material hardening which can be detected via hardness measurements [31]. Based on this assumption, the nano-hardness measurements near the cutting edge were performed for samples obtained from the rings with four different cutting clearances. The distribution of nano-indentation measurements near the cutting edge of four different samples before annealing is presented in Figure 7.

**Figure 8:**
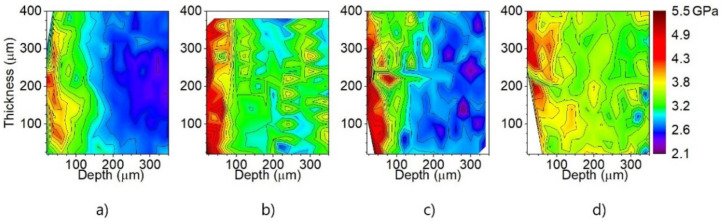
Maps of hardness obtained by means of nanoindentation measurements near the cutting edge before annealing of rings with cutting clearances corresponding to 1% (**a**), 3% (**b**), 5% (**c**), 7% (**d**).

The measurement was performed in a 20 µm pitch in both a parallel and perpendicular plane to the specimen in 22 parallel rows spaced by 20 µm, see Figure 7. The coordinates of the indentation were measured from the cutting edge in each row. The ratio of nanoindentation hardness in each measured point and away from the shear area was determined as the work hardening intensity. The hardening intensity was determined by the measuring position and the cutting clearance. The distribution of nano hardness intensity near the cutting edge before annealing is shown in Figure 8. These graphs demonstrate similar results which were obtained by means of local misorientation maps, see Figure 5. As one can see, the maximum value of hardness was obtained in the region vicinity of the cutting edge with a width of approximately 100 µm.

### 3.4. Analysis of Magnetic Loss

**Figure 9:**
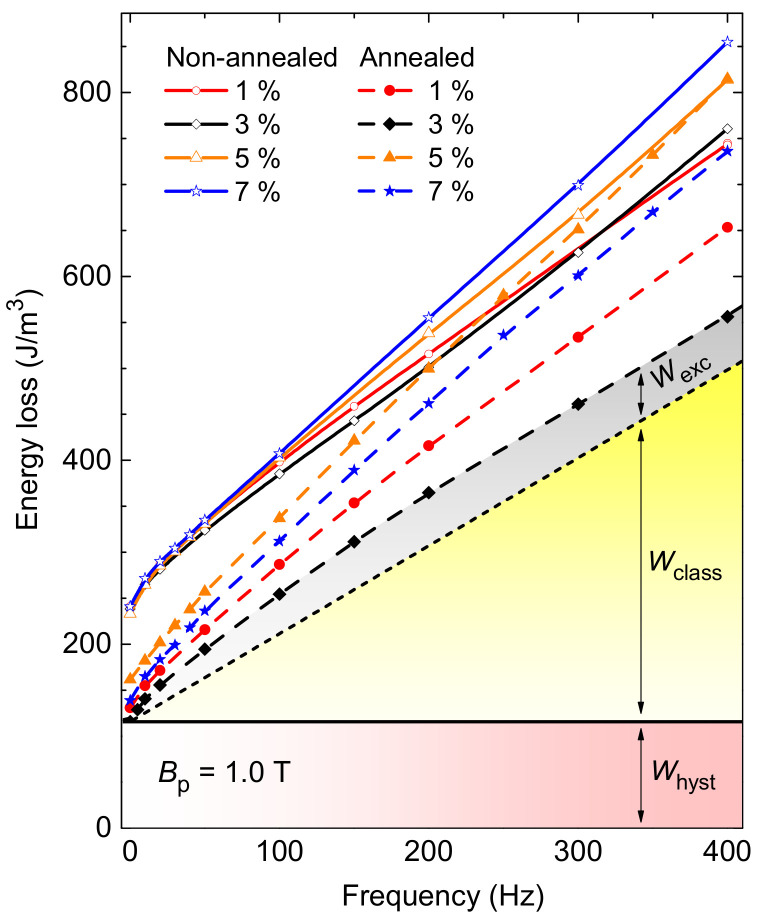
Energy loss behaviour versus magnetizing frequency at *B*_p_ = 1.0 T in NO electrical steels punched with different cutting clearances before and after annealing. Example of loss decomposition into hysteresis, classical, and excess loss components is provided for annealed steel sample with a cutting clearance of 3% showing the most efficient loss response.

The influence of the energy loss, *W(f)*, on the frequency up to 400 Hz at *B*_p_ = 1.0 T for Fe–Si punched samples with different cutting clearance is shown in Figure 9. In non-annealed samples, the minimum loss response is exhibited using the 3% cutting clearance tool up to the crossover (about 300 Hz) with the sample of 1% clearance. The progressive increase of the overall loss dependence in the samples with 5% and 7% clearance is observed. The dynamic heat treatment results in the core loss reduction by ~55% under quasi-static condition in all the samples. Significant loss suppression in the annealed sample of 3% clearance is preserved at all frequencies, while the *W*(*f*) of the sample 5% clearance after annealing attains its behaviour in non-heat-treated case on entering the highest frequencies. A presence of the minimum in the loss figure versus cutting clearance is supported by recent studies of blanked edge quality [26,32]. The total loss in annealed samples at the frequency of 50 Hz and *B*_p_ = 1.0 T can be suppressed in the studied case by 23% by adjusting the cutting clearance (Figure 9, cutting clearance of 5% versus 3%).

**Figure 10:**
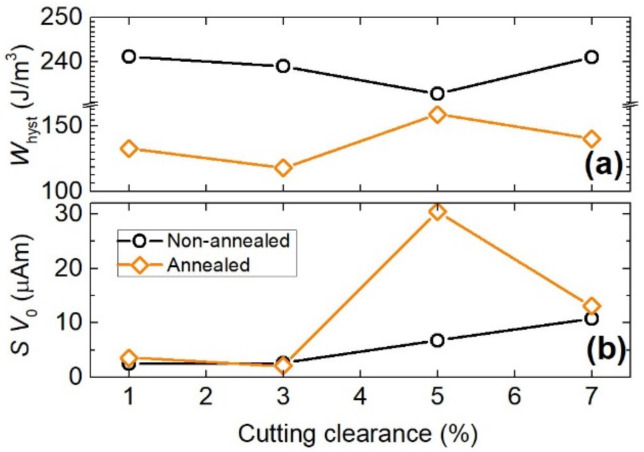
Comparison of (**a**) hysteresis loss and (**b**) statistical quantity *SV*_0_ in the investigated steels before and after annealing. (**a**) is obtained experimentally and (**b**) is predicted theoretically.

Separation of the energy loss into the three components of hysteresis *W*_hyst_, classical *W*_class_, and excess *W*_exc_ loss—i.e., *W*(*f*) = *W*_hyst_ + *W*_class_(*f*) + *W*_exc_(*f*)—is a direct consequence of the physical analysis of stochastic magnetization process from the statistical point of view looking at the eddy current associated dissipation on the scale of strongly localized Barkhausen jumps, macroscopic sample’s cross-section, and evolving domain walls, respectively [33]. An example of the loss decomposition in the annealed steel with the cutting clearance of 3% is illustrated in Figure 9. Hysteresis loss, determined by measurement under quasi-dc condition and provided in Figure 10a, shows a faint minimum at the clearance of 5% in non-annealed materials while it shows the same at 3% in annealed materials. The clearance of 5% in non-annealed samples is therefore considered to have the smallest detrimental effect on the structure of the steel due to deformation and consequent introduction of additional pinning sites hindering the domain wall motion. The authors in [34] observed the most significant influence of cutting clearance on hysteresis loss component. In the case of homogeneous flux penetration—i.e., in lacking skin-effect conditions—the classical loss component in a lamination of thickness *d* and electrical conductivity *σ*, is given as

(1) $$W_{\mathrm{class}}\left(B_{p},f\right)=\frac{\pi }{6}\sigma d^{2}B_{p}^{2}f$$

Since the electrical conductivity of the specimens is not affected by cutting procedure, the classical loss contributes to the measured one by the same proportion in all the materials (as in Figure 9). The significant changes in *W*(*f*) behaviour are only due to static and dynamic loss contributions associated to the domain walls. Following the loss separation concept, the excess loss due to eddy currents localized around the evolving domain walls is derived as *W*_exc_(*B*_p_, *f*) = *W*(*B*_p_, *f*) − *W*_hyst_(*B*_p_) − *W*_class_(*B*_p_, *f*).

**Figure 11:**
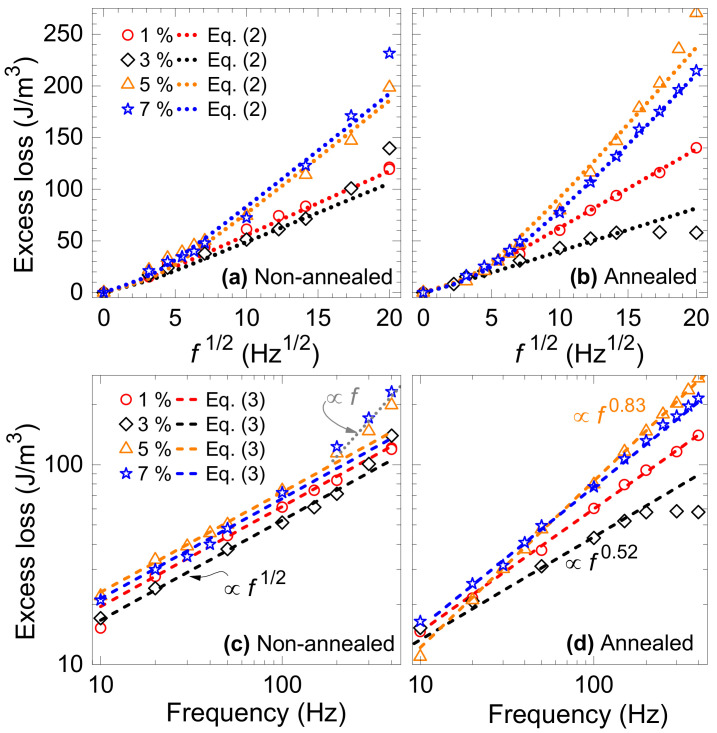
Excess loss in (**a**,**c**) non-annealed and in (**b**,**d**) annealed electrical steels of different cutting clearance. Experimentally obtained loss (points) are compared with its theoretical predictions represented by dotted lines calculated through Equation (2) in case of (**a**,**b**) and by short-dashed lines through Equation (3) in case of (**c**,**d**).

In the statistical theory of losses, the general formulation of *W*_exc_ is based on the concept of the statistics of magnetic objects (m.o.s) by which the magnetization reversal is accomplished. Each m.o. corresponds to the region of a domain wall or an ensemble of neighboring domain walls moving in a correlated fashion [35]. Considering the uniformly distributed pinning fields in the cross-section *S* with the quantity *SV*_0_ related to the width of the distribution and the normalized number of reversing m.o.s *n*_0_/*S* per unit area under quasi-static conditions, the complete form of excess loss predicted under the sinusoidal induction profile is

(2) $$W_{\mathrm{exc}\;}\left(B_{p},f\right)=2n_{0}V_{0}B_{p}\int_{0}^{\pi /2}\left[{\left(1+\frac{8\pi f\sigma GSV_{0}}{n_{0}^{2}V_{0}^{2}}B_{p}\cos\phi \right)}^{1/2}-1\right]\cos\phi \;d\phi$$

where *G* = 0.1356, *B*_p_ is the peak magnetic induction, *σ* is the electrical conductivity, *n*_0_ and *V*_0_ are two statistical parameters related to the distribution of the local threshold fields for magnetization reversal across the sample cross-section, and *Φ* comes from the integration over one period of magnetization cycle [36]. Calculated excess loss is compared with the experimentally obtained value and plotted against *f*^1/2^ at *B*_p_ = 1 T in the materials of different cutting clearance in Figure 11 before annealing (a) and after annealing (b). The values of quantity *SV*_0_ used in Equation (2) to predict *W*_exc_ are plotted versus cutting clearance in Figure 10b. To note the similar qualitative behaviour of *W*_hyst_ and statistical quantity *SV*_0_ in annealed materials (Figure 10a,b). *SV*_0_ determines the span of local coercive fields activating switching of m.o.s which are at the core of hysteresis loss origin [37]. An excellent agreement in comparison in Figure 11a,b is observed from quasi-dc regime up to the frequency of 200 Hz, beyond which the deviation from the law projected by Equation (2) occurs as the fingerprint of incipient skin-effect. The flux penetration on entering higher magnetizing frequencies becomes inhomogeneous and Equation (1) does not provide adequate description of the classical eddy currents. According to the approach in [38], by resorting to the formulation of magnetic constitutive relation in terms of complex permeability, one can describe the classical loss even at frequencies affected by skin effect. This is inevitable on entering the magnetizing frequencies in the kilohertz range even at low peak inductions [39]. Besides this high-frequency deviation in the case of annealed steels, the overall changes in the slope of the experimental *W*_exc_(*f*) versus cutting clearance are observed (cf. Figure 11b,d in different frequency representation).

The loss behaviour different from *W*_exc_ ∝ *f*^1/2^ well inside the skin-effect-free region suggests the distortion of the uniform distribution of local pinning fields *Γ*(*H*_c_) [40]. Considering the non-uniform distribution of the type *Γ*(*H*_c_) ∝ *H*_c_*^n^*^−1^ with 0 < *n* ≤ 1 (reducing to the uniform distribution for *n* = 1) and the fundamental correlation between hysteresis and excess loss, the generalized expression translating the *W*_hyst_ into *W*_exc_, can be worked out as

(3) $$W_{\mathrm{exc}}\left(B_{p},f\right)=4{\left[\beta_{\mathrm{eddy}\;}W_{\mathrm{hyst}}^{n}\left(B_{p}\right)\;\langle s\rangle \;\left(B_{p}/B_{s}\right)\;f\right]}^{1/\left(n+1\right)}$$

where *B*_p_ is the peak magnetic induction, $\beta_{\mathrm{eddy}}=4\sigma GB_{s}^{2}\langle s\rangle$ is the eddy current related damping coefficient, *G* = 0.1356, *σ* is the electrical conductivity, *B*_s_ is the saturation induction, $\langle s\rangle$ is the mean size of grain in which the domain wall generated magnetization reversal takes place, *f* is the magnetizing frequency, and 0 < *n* ≤ 1 [41]. The general validity of Equation (3) allows to use it also for nearly insulating materials—e.g., soft ferrites—by simple change of *β*_eddy_ by spin damping *β*_sd_ coefficient describing the major dissipation channel in such materials [41]. Mean grain size $\langle s\rangle$ is shown in Figure 4 as determined from the micrographs of grain structure. Figure 11c,d provide a comparison of experimentally determined and theoretically predicted *W*_exc_ via Equation (3). The excess loss figure shown in Figure 11c for the samples before annealing is well described by the uniform distribution *Γ* with some deviations at the highest frequencies by changing the slope of *W*_exc_ ∝ *f^q^* from *q* = 1/2 to *q* = 1. *W*_exc_ in annealed specimens, however, follow the loss predicted by Equation (3) using the parameter *n* varying between 0.20–0.92 corresponding to the changing power coefficient from *q* = 0.83 to *q* = 0.52. While the distribution of the local coercive fields in the sample of 3% cutting clearance with the lowest *W*(*f*) response is quite close to the uniform one—i.e., *Γ*(*H*_c_) ∝ *H*_c_^−0.08^ ≈ 1—the anomalous increase of *W*(*f*) in the annealed samples of higher cutting clearance at high frequencies can be understood in terms of non-uniform distribution function *Γ*(*H*_c_) ∝ *H*_c_^−0.8^. It is supported by the equivalent behaviour found in soft magnetic alloys and ferrites [40].

## 4. Conclusions

- The energy loss response of the non-oriented Fe-Si electrical steels can be favourably affected by lower cutting clearance and subsequent annealing.
- Fine-grained microstructure near the cutting edge is the most important structural parameter responsible for increasing magnetic loss with higher cutting clearance.
- The excess loss is found as the most sensitive loss component to cutting clearance and its magneto-structural correlation is quantified by uniform distribution of local pinning fields in non-annealed steels which evolves to non-uniform distribution after annealing.

## Data Availability

The data presented in this study are available on request from the corresponding author.
